# Assessing donor-recipient arterial pressure dynamics in STA-MCA bypass for moyamoya disease

**DOI:** 10.1186/s41016-024-00367-2

**Published:** 2024-05-12

**Authors:** Mohamed Helmy, Yujun Liao, Zehao Zhao, Zhiqi Li, Kangmin He, Bin Xu

**Affiliations:** 1 Neurosurgery Department, Fudan University Huashan Hospital, Shanghai, 200040 People’s Republic of China; 2 National Center for Neurological Disorders, Shanghai, 200040 People’s Republic of China

**Keywords:** Moyamoya disease, Pressure gradient, Cerebral hemodynamic, Suzuki stage

## Abstract

**Background:**

In bypass surgery for moyamoya disease (MMD), the superficial temporal artery’s (STA) pressure needs to surpass that of the cortical M4 recipient of the middle cerebral artery (MCA), boosting cerebral blood flow into the MCA and enhancing cerebral circulation. This study investigates the STA-MCA arterial pressure parameters and gradients during bypass surgery, aiming to deepen our understanding of hemodynamic shifts pre- and post-operation.

**Methods:**

DSA imaging data were prospectively collected from patients diagnosed with bilateral MMD who underwent STA-MCA bypass surgery between 2022 and 2023 and stratified according to the Suzuki stage. The mean arterial pressure (MAP) of the donor and recipient arteries was directly measured during the STA-MCA bypass procedure, and these data were statistically analyzed and evaluated.

**Results:**

Among 48 MMD patients, Suzuki grading revealed that 43.8% were in early stages (II and III), while 56.2% were in advanced stages (IV, V, and VI). Predominantly, 77.1% presented with ischemic-type MMD and 22.9% with hemorrhagic type. Pre-bypass assessments showed that 62.5% exhibited antegrade blood flow direction, and 37.5% had retrograde. The mean recipient artery pressure was 35.0 ± 2.3 mmHg, with a mean donor-recipient pressure gradient (δP) of 46.4 ± 2.5 mmHg between donor and recipient arteries. Post-bypass, mean recipient artery pressure increased to 73.3 ± 1.6 mmHg. No significant correlation (*r* = 0.18, *P* = 0.21) was noted between δP and Suzuki staging.

**Conclusion:**

Our study elucidated that cerebral blood pressure significantly decreases beyond the moyamoya network at the distal M4 segment. Furthermore, we observed bidirectional flow in MCA territories and a significant positive pressure gradient between the STA and M4 segments. The lack of correlation between Suzuki stages and M4 pressures indicates that angiographic severity may not reflect hemodynamic conditions before surgery, highlighting the need for customized surgical approaches.

## Background

Moyamoya disease (MMD) is a chronic cerebrovascular disorder characterized by progressive stenosis or occlusion of the terminal segment of the internal carotid artery (ICA) and its branches, manifesting as bilateral or unilateral vasculopathy [1, 2]. These pathophysiological changes significantly disrupt cerebral hemodynamics and can lead to severe consequences such as ischemic or hemorrhagic events [3, 4]. In MMD patients, the emergence of smoky-like vessels near the skull base is a compensatory mechanism triggered by ischemic hypoxia, representing collateral circulation at early stages [5, 6]. These moyamoya vessels are a physiological response to the altered cerebral hemodynamics [7], associated with changes in blood flow dynamics and pressure gradients within the cerebral vasculature.

As blood flow traverses through the moyamoya vessels, the increased surface contact area, heightened friction, and turbulence lead to significant energy loss, impacting the velocity and pressure of the blood flow [8], and the mean transit time through the distal M3–M4 network extends. These dynamics are aptly explained by the throttle principle in fluid mechanics, aligning with findings from CT perfusion studies in moyamoya patients [9]. Despite these insights, the precise pressure levels as blood traverses through the moyamoya vessel to reach the M4 segment remain unknown. Our study aims to measure the pressure within the distal M4 segment of the MCA to quantify this reduction in pressure and elucidate its implications for cerebral hemodynamics.

The STA-MCA bypass of moyamoya disease is considered to be a bypass that increases cerebral blood flow, that is, the pressure difference between STA and M4 is positive so that blood flow can pass from the external carotid artery system into the internal carotid artery system, providing more blood flow to the cortex. According to the Hagen-Poiseuille law, blood flow is directly proportional to the pressure difference, and the pressure difference is the only dynamic factor that drives blood flow into the cortex. Our previous studies have shown that a parallel bypass that aligns the blood flow of the donor vessel with the direction of the blood flow to the recipient vessel is more reasonable. However, we do not have actual data on the proportion of the inverse MCA pressure gradient and the specific M4 pressure.

Traditionally, it was assumed that hemodynamic stress in MMD correlated with the severity of its cerebral vasculopathy. The widely utilized Suzuki grading system classifies MMD angiographic severity into six stages [10, 11], ranging from evident stenosis at the distal internal carotid artery bifurcation in Stage I to complete loss of blood supply from both anterior and posterior cerebral circulation in Stage VI. Although some studies have explored the correlation between Suzuki grading and cerebral hemodynamics [12, 13], this grading system primarily offers a morphological description. It fails to capture the relationship between angiographic severity and clinical presentation due to complex anatomical and physiological variations [14–16].

Establishing a favorable pressure difference between the donor STA and the M4 MCA branch is vital for the success of the bypass procedure. Through our investigation, we seek to establish whether there is a significant correlation between the pressure difference created by the bypass and the Suzuki stages of MMD, which could provide insights into optimizing surgical approaches for enhancing cerebral perfusion in moyamoya patients.

## Methods

### Patient selection and stratification

Following approval from our hospital’s Institutional Review Board (IRB) and obtaining written, informed consent from each study patient, we prospectively enrolled 50 patients diagnosed with MMD who underwent bypass surgery at our institution between June 2022 and June 2023. A thorough preoperative assessment utilizing digital subtraction angiography (DSA) was conducted on all patients. Two MMD patients who exhibited aneurysms on DSA in the ipsilateral proximal segment of the MCA were excluded from the study [17] in order to mitigate potential pressure variations in cortical arteries due to these vascular anomalies [18]. Subsequently, the remaining cohort of 48 eligible MMD patients was stratified according to the Suzuki grading system [11] and revised by senior authors.

The Suzuki classification system, utilized for evaluating MMD severity, is predicated on angiographic observations and subdivided into six distinct stages. In Stage I, stenosis is present at the carotid bifurcation. Progressing to Stage II, the steno-occlusive changes at the carotid bifurcation become more pronounced, accompanied by the emergence of basal moyamoya vessels. Stage III is characterized by a significant proliferation of moyamoya vessels and leptomeningeal collateralization originating from the posterior cerebral arteries. Transitioning to Stage IV, there is a reduction in moyamoya vessels alongside continued occlusive changes in the basal arteries. Stage V is delineated by a complete occlusion of the internal carotid arteries and the proximal segments of the middle cerebral arteries, coupled with a marked development of collateral vasculature. Finally, Stage VI is defined by an exhaustive obstruction and cessation of blood supply via the internal carotid arteries, complemented by an extensive expansion of collateral vessels from the external carotid artery (ECA).

**Fig. 1:**
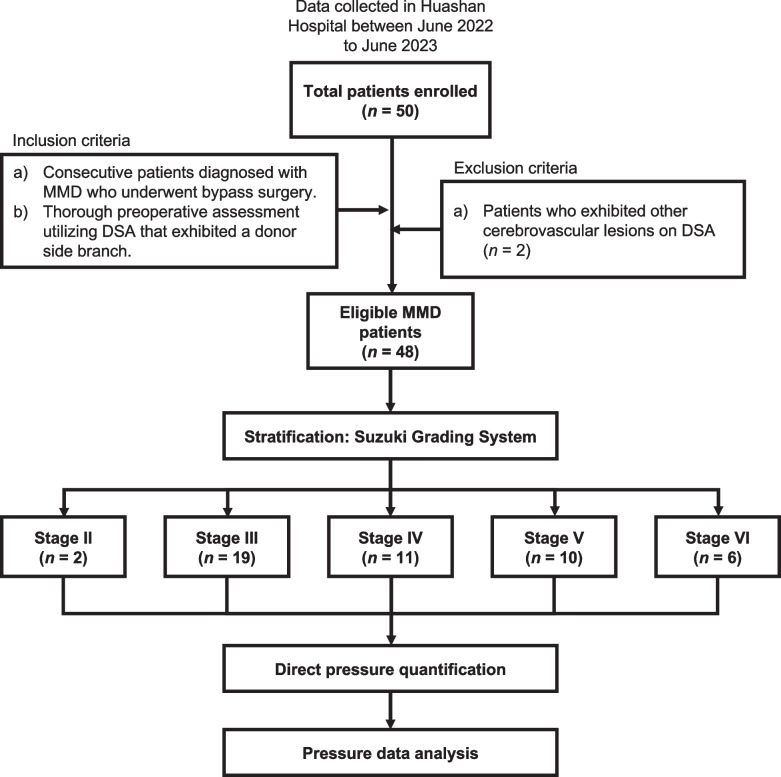
Flowchart of the study methodology for moyamoya disease (MMD) enrolled in the study. DSA, digital subtraction angiography

In some patients with bilateral MMD, both cerebral hemispheres might demonstrate different Suzuki stages. In this study, the Suzuki stage of each operated hemisphere was categorized separately [11] with the distribution as follows: 2 patients with stage II, 19 with stage III, 11 with stage IV, 10 with stage V, and 6 with stage VI. All individuals underwent unilateral STA-MCA bypass surgery. Fig. 1 shows the flow chart of the study methodology.

### Pressure measurement method

Vessel preparation for pressure measurement involved a systematic dissection of the donor artery and its accompanying side branch, followed by preparation of the donor STA branch for microscopic end-to-side anastomosis. After completing the anastomosis, Abbott’s wireless PressureWire^™^ X Guidewire (Plymouth, MN, USA) was inserted into a donor-side branch to facilitate measurements from various segments of both the donor and recipient arteries.

**Fig. 2:**
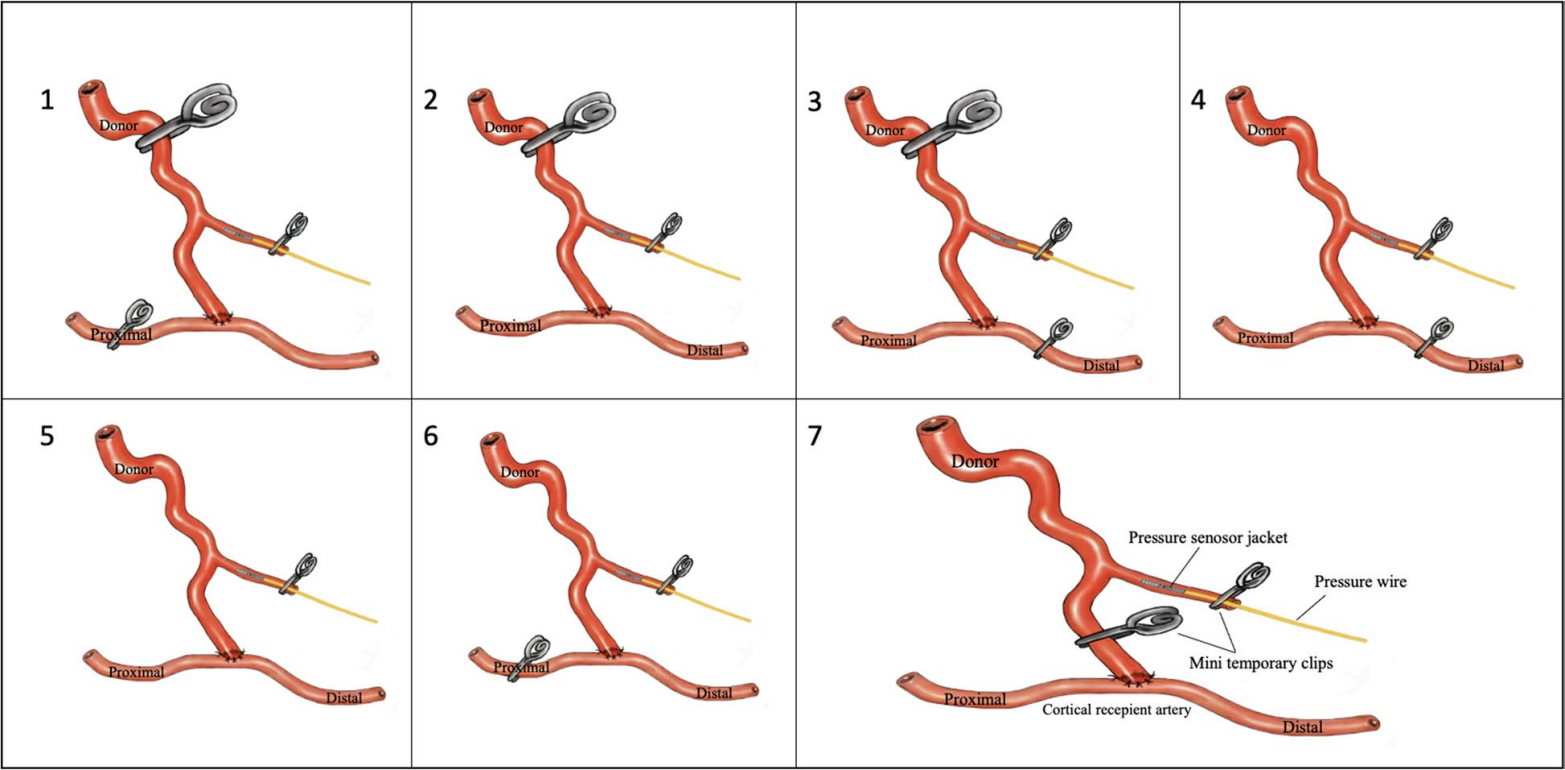
Schematic diagram showing the pressure parameters utilized during STA-MCA (P1–P7). P1–3 represented the pre-bypass cortical recipient artery (P2) and its segments (distal, P1 and proximal, P3) pressure, while the donor artery was clipped by a temporary clip; P4–6 represents the post-bypass cortical recipient artery (P5) and its segment (proximal, P4 and distal, P6) pressure. The methodology for obtaining the intraoperative donor artery pressure is depicted in P7

The methodology for pressure measurement was interwoven into the surgical procedure, employing a step-by-step approach represented by seven pressure parameters (P1 to P7), as depicted in Fig. 2. The procedure entails recording seven pressure metrics from both donor and recipient arteries post-anastomosis. Initially, the first mean arterial pressure (MAP) measurement (P1) is acquired from the recipient artery’s distal segment, with temporary clips on both the donor and the proximal segment of the recipient (Fig. 2 (1)). The second measurement is conducted with the recipient segments unclipped and only the donor artery proximally clipped (Fig. 2 (2)). The third measurement (P3) captures the MAP of the recipient’s proximal segment, while the donor and the recipient’s distal segment are clipped (Fig. 2 (3)). For the subsequent measurements, the temporary clip on the proximal donor is removed, maintaining the recipient’s clipping as per P3, to record the pressure at the junction of the donor and proximal recipient segment (P4) (Fig. 2 (4)). Removing the clip from the distal recipient allows for a pressure recording with all bypass pathways open (P5) (Fig. 2 (5)). The next step (P6) involves clipping the proximal segment of the recipient alone (Fig. 2 (6)). The final pressure metric is derived from the donor artery, post-occlusion distal to the side branch (Fig. 2 (7)). This ordering system minimizes the temporary occlusion time during the measurement.

This deliberate approach allowed for accurately assessing the MAP and pressure gradients (δP) between the donor and the cortical recipient artery segments. While the anastomosis was performed and the main donor artery was clipped, all obtained pressure data was referred to as pre-bypass pressure (Fig. 2, P1–P3, P7). Conversely, when the main donor artery clip was removed (Fig. 2, P4–P6), the pressure was referred to as post-bypass pressure. A comprehensive elaboration of the entire measurement procedure is available in our recent publication [19]. The intraoperative MAP of the radial artery measured via the anesthesiologist’s monitoring system was recorded and compared with the MAP of the STA as a donor branch for further analysis.

### Statistical analysis

Continuous variables adhering to a normal distribution were expressed as mean ± standard error of the mean (mean ± s.e.m). In contrast, the median and interquartile range (IQR) represented those deviating from normal distribution. Categorical data were summarized using counts and corresponding percentages.

Given the limited sample size of Stage II patients (*n* = 2), precluding the application of normality tests, nonparametric analyses including the Kruskal-Wallis test and Dunn’s multiple comparisons test were employed to assess disparities in arterial pressure alterations and other demographic or clinical parameters across Suzuki stages. The chi-square test (*χ*^2^) or Fisher’s exact test was applied appropriately for categorical variables. Pearson’s or Spearman’s correlation analysis was employed to investigate the associations among variables. The two-tailed paired *t*-test was employed to evaluate the differences between two paired continuous variables whose difference is normally distributed, such as pre-and post-bypass recipient artery pressure for Stages III–VI patients. In contrast, nonparametric Wilcoxon matched-pairs signed-ranks test was employed to evaluate the differences between two paired continuous variables for Stage II patients (*n* = 2).

Throughout the analysis, significance was determined at a threshold of *P* < 0.05. All computational and statistical procedures were executed using R software (version 3.6.3), Stata/SE (version 15.1), or GraphPad Prism (version 8.4.0).

## Results

### Patient demographics and clinical overview

Baseline demographics and clinical characteristics of the study participants are shown in Table 1. Among the 48 MMD patients, a relatively equal sex distribution was observed, with 52.1% male and 47.9% female. The average age was 47.5 ± 1.6. For Suzuki grading, 43.8% were categorized as early stage (II and III) and 56.2% as advanced stage (IV, V, and VI). Moreover, 77.1% of all patients presented with ischemic-type MMD, while 22.9% presented with hemorrhagic type. Right-sided STA-MCA bypass was performed on 52.1% of patients and left-sided bypass on 47.9%. Patients in stages V and VI had significantly higher mean ages (54 and 53 years, respectively) than those in stages II, III, and IV (40, 48, and 40 years, respectively). A statistically significant age distribution difference was noted between early and advanced Suzuki stages (*P* = 0.045). Table 2 shows the patients’ baseline demographic characteristics and clinical assessments stratified by the Suzuki stage.

**Table 1:** Clinical characteristics of study patients

**Parameter**	**Total (*n* = 48)**	**Percentage (%)**
**Age, years (mean ± s.e.m.)**	47.5 ± 1.6	
**Sex, no.**		
Male	25	52.1
Female	23	47.9
**Suzuki staging, no.**		
Early stage	2	4.2
II		
III	19	39.6
Advanced stage		
IV	11	22.9
V	10	20.8
VI	6	12.5
**Clinical presentation, no.**		
Ischemic	37	77.1
Hemorrhagic	11	22.9
**Surgical site, no.**		
Right	25	52.1
Left	23	47.9
**Pre-bypass recipient flow direction, no.**		
Anterograde	30	62.5
Retrograde	18	37.5

**Table 2:** Baseline demographic characteristics and clinical assessments of patients stratified by Suzuki stage (*n* = 48)

**Parameters**	**Suzuki staging (II–VI)**					**Total**	***p*-value**
	**II (*n* = 2)**	**III (*n* = 19)**	**IV (*n* = 11)**	**V (*n* = 10)**	**VI (*n* = 6)**		
**Age, years (mean ± s.e.m.)**	40.0 ± 5.0	47.6 ± 2.2	40.0 ± 3.8	54.0 ± 2.8	53.0 ± 4.0	47.5 ± 1.6	0.045*
**Clinical presentation, no.**							
Ischemic	2	14	8	7	6	37	
Hemorrhagic	0	5	3	3	0	11	0.574
**Intraoperative radial artery pressure, mmHg (mean ± s.e.m.)**							
	80.0 ± 0.0	84.4 ± 2.3	83.1 ± 2.7	84.8 ± 3.5	94.7 ± 5.4	85.3 ± 1.5	0.382
**Donor artery pressure, mmHg (mean ± s.e.m.)**							
	77.0 ± 1.0	78.3 ± 2.7	82.2 ± 3.1	82.8 ± 2.7	92.3 ± 5.5	81.8 ± 1.6	0.191
**Pre-bypass recipient pressure, mmHg (mean ± s.e.m.)**							
Proximal segment	34.5 ± 18.5	37.3 ± 3.1	25.4 ± 3.2	38.1 ± 7.8	40.7 ± 5.1	35.0 ± 2.4	0.179
Distal segment	33.0 ± 7.0	36.1 ± 3.6	21.0 ± 3.0	31.1 ± 5.5	37.2 ± 5.0	31.6 ± 2.2	0.061
Entire recipient artery	34.5 ± 15.5	36.9 ± 3.0	25.4 ± 3.1	38.0 ± 7.7	41.5 ± 5.0	35.0 ± 2.3	0.158
**Post-bypass recipient pressure, mmHg (mean ± s.e.m.)**							
Proximal segment	60.0 ± 0.0	73.3 ± 2.2	74.5 ± 2.2	71.6 ± 3.7	86.3 ± 4.3	74.3 ± 1.6	0.029*
Distal segment	56.5 ± 4.5	74.4 ± 2.5	75.5 ± 2.9	73.7 ± 2.9	88.3 ± 5.0	75.5 ± 1.7	0.038*
Entire recipient artery	58.0 ± 3.0	72.2 ± 2.4	73.5 ± 2.7	71.3 ± 3.6	85.3 ± 3.4	73.3 ± 1.6	0.024*
**δP between donor and recipient in pre-bypass state, mmHg (mean ± s.e.m.)**							
	42.5 ± 14.5	41.4 ± 3.6	56.8 ± 4.0	42.7 ± 6.7	50.8 ± 7.0	46.4 ± 2.5	0.222

^*^P < 0.05

### Blood flow direction and radial/donor pressure relationship

**Fig. 3:**
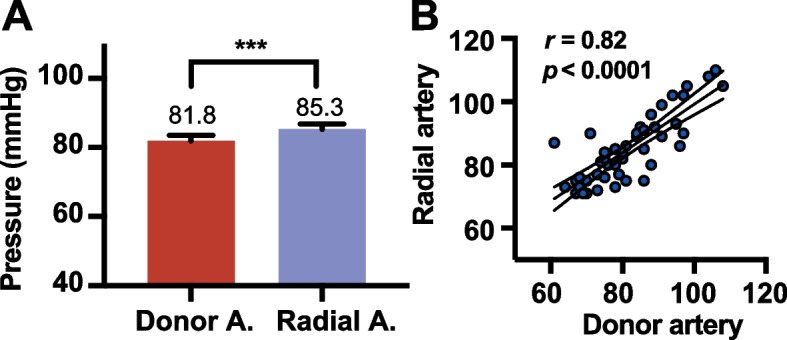
Intraoperative pressures of the donor and radial arteries prior to STA-MCA bypass. **A** The bar chart illustrates the intraoperative pressures of the donor and radial arteries, represented as the mean ± standard error of the mean (s.e.m.). Donor A., donor artery; radial A., radial artery; ***P < 0.001. **B** The scatter plot illustrates the strong positive correlation between the pressures of the donor and radial arteries (Pearson correlation coefficient *r* = 0.82, *P* < 0.0001)

At pre-bypass, 62.5% of patients exhibited antegrade blood flow, while the remaining 37.5% displayed retrograde blood flow. Intraoperatively, the mean radial artery pressure was 85.3 ± 1.5 mmHg, while the mean donor artery pressure was 81.8 ± 1.6 mmHg, with a mean pressure difference of 3.4 ± 0.94 mmHg (Fig. 3).

### Pre-bypass recipient pressure assessment

Pre-bypass arterial pressure readings from the cortical recipient segments were obtained and analyzed (Fig. 2, P1–P3). The recipient artery exhibited a mean pressure of 35.0 ± 2.3 mmHg. Upon further analysis, the proximal segment of the recipient artery displayed a mean pressure of 35.0 ± 2.4 mmHg. Conversely, the distal segment of the recipient artery had a mean pressure of 31.6 ± 2.2 mmHg. Notably, the mean pressure gradient (δP) between the donor and recipient artery was 46.4 ± 2.5 mmHg.

### Post-bypass recipient pressure assessment

**Fig. 4:**
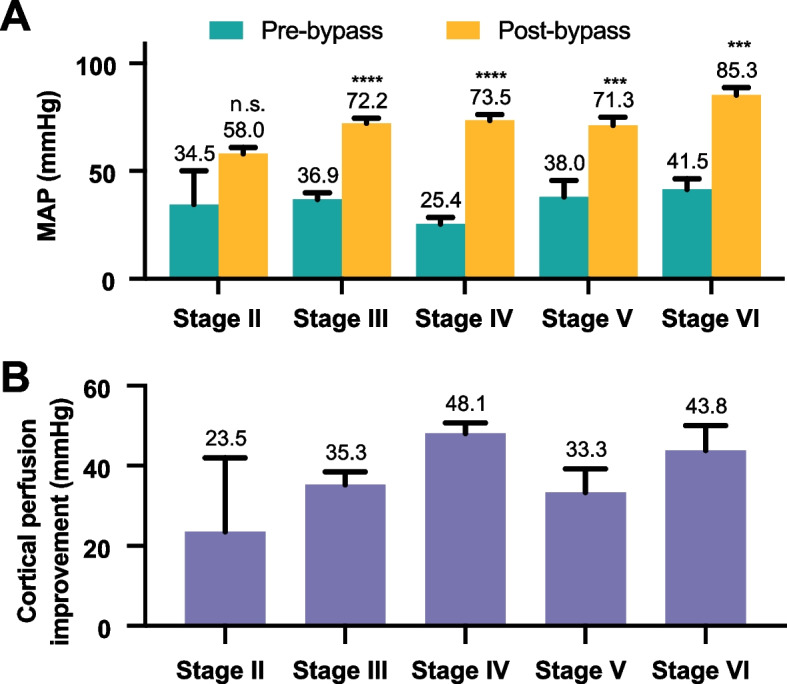
Mean arterial pressure (MAP) differences of recipient artery pre- and post-STA-MCA bypass for each Suzuki stage. **A** The bar chart shows the MAP differences of recipient artery pre- and post-STA-MCA bypass for each Suzuki stage (mean ± s.e.m.). ***P < 0.001; ****P < 0.0001; n.s., not significant, compared to the pre-bypass MAP, Wilcoxon matched-pairs signed-ranks test for Stage II, paired *t*-test for Stages III–VI. **B** The bar chart illustrates the improvement in cortical perfusion during bypass procedures, specifically the post-bypass increases in MAP in recipient arteries for each Suzuki stage (mean ± s.e.m.)

Upon release of the temporary clip on the proximal donor artery (Fig. 2, P4–P6), a substantial change was observed in the mean pressure of the recipient artery, registering at 73.3 ± 1.6 mmHg. Further examination of the proximal and distal segments revealed mean pressures of 74.3 ± 1.6 mmHg and 75.5 ± 1.7 mmHg, respectively. The corresponding pressure changes in the recipient artery pre- and post-bypass are depicted in Fig. 4A. The improvement of cortical recipient perfusion among Suzuki stages II, III, IV, V, and VI was 23.5 ± 18.5, 35.3 ± 3.2, 48.1 ± 2.6, 33.3 ± 5.9, and 43.8 ± 6.3 mmHg, respectively (Fig. 4B). However, no significant relationship (*r* = 0.17, *P* = 0.25) was identified between the improvement of cortical recipient perfusion and Suzuki staging.

### Relationship between donor-recipient pressure gradient and Suzuki stages

**Fig. 5:**
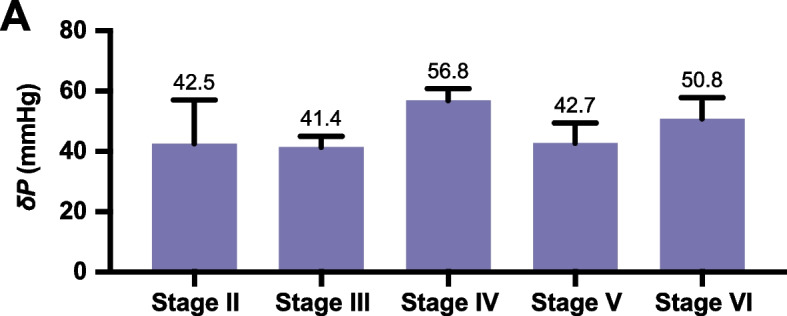
Mean pressure gradient (δP) between the donor and recipient arteries for each Suzuki stage. **A** The bar chart depicting the mean pressure gradient (δP) between the donor and recipient arteries for each Suzuki stage represents the mean ± s.e.m

There was no significant relationship (*r* = 0.18, *P* = 0.22) between δP and Suzuki staging. The mean δP among Suzuki stages II, III, IV, V, and VI were 42.5 ± 14.5, 41.4 ± 3.6, 56.8 ± 4.0, 42.7 ± 6.7, and 50.8 ± 7.0 mmHg, respectively (Fig. 5).

## Discussion

For the first time, our study quantitatively assesses the pressure parameters and gradient between the donor and recipient arteries during STA-MCA bypass surgery in MMD. This study highlights a vital, yet previously neglected, aspect of cerebral hemodynamics in MMD and its potential correlation with disease severity. By applying Poiseuille’s law, we underscore the pivotal role of pressure gradients on cerebral blood flow [20]. These findings emphasize the integration of comprehensive hemodynamic evaluations alongside traditional angiography in STA-MCA bypass decision-making processes.

Contrary to conventional expectations, our study found no significant correlation (*p*-value = 0.236) between the Suzuki stage and the pre-bypass pressure in the recipient cortical arteries. Interestingly, across Suzuki stages II to VI, we observed a range of mean pre-bypass recipient artery pressures, 34.5 ± 15.5 mmHg, 36.9 ± 3.0, 25.4 ± 3.1, 38.0 ± 7.7, 41.5 ± 5.0, and 35.0 ± 2.3, respectively. This challenges the traditional view that associates higher Suzuki stages with more severe clinical manifestations, primarily due to presumed lower perfusion levels [16, 21, 22], indicating that hypoperfusion concerns are not limited to advanced stages but are also present in early stages of MMD. Our data underscores the complexity of this disease beyond mere angiographic severity.

Post-bypass, a noteworthy improvement in recipient artery pressures across all stages was observed (II to VI: 58.0 ± 3.0 mmHg, 72.2 ± 2.4 mmHg, 73.5 ± 2.7 mmHg, 71.3 ± 3.6 mmHg, and 85.3 ± 3.4 mmHg, respectively), reinforcing the efficacy of direct bypass surgery in enhancing cortical perfusion, independent of the disease’s angiographic stage [23]. In all patients, the mean recipient artery pressure across all stages reached 73.3 ± 1.6 mmHg. This finding aligns with standard cerebral perfusion pressure values [24].

Remarkably, despite the absence of a statistically significant difference in the pressure gradient (δP) between donor and recipient arteries across various Suzuki stages (*p*-value = 0.222), with an average δP recorded at 46.4 ± 2.5 mmHg, variations in recipient artery pressure were discernible within the same Suzuki stage. Predominantly, the sites of anastomosis were located in the M4 branches at the posterior distal area of the Sylvian fissure. This observation can be attributed to the consistency in the variance of pre-bypass pressures between the recipient and donor arteries across the stages. It also highlights the STA-MCA bypass surgery’s capacity to immediately improve cerebral perfusion pressures across the spectrum of MMD, thereby offering immediate hemodynamic benefits [25, 26].

The study also revisits the arterial pressure dynamics between the donor STA and the systemic circulation. Consistent with the findings of Shulgina et al., we also observed that the mean arterial pressure in the donor STA was 3.4 mmHg marginally lower than the systemic blood pressure recorded via intraoperative radial artery monitoring [27]. This supports the feasibility of predicting mean donor artery pressure in STA-MCA bypass patients without the need for direct measurement. This finding prompts a reevaluation of the STA’s role as a “low-flow” donor, suggesting instead that with strategic selection of the anastomosis site—guided by a positive donor-recipient δP—optimal perfusion improvements can be achieved.

The complexity of blood flow dynamics in MMD was exemplified by the presence of both antegrade and retrograde flows in the cortical MCA. Among our cohort, 62.5% of patients exhibited antegrade cortical MCA flow at the pre-bypass setting, while 37.5% showed retrograde flow in cortical MCA. Notably, retrograde flow was observed across both early and advanced Suzuki stages, possibly due to reduced proximal pressure in early-stage and advanced-stage collateral development [28]. Subsequently, this underscores the importance of individualized surgical planning, which should be informed by preoperative analysis of flow direction in DSA images to choose the area with the lower pressure in cortical recipient (downstream area) [29, 30] to create a bypass with a positive δP which can be assessed by observing the progression of the contrast agent through the MCA [31, 32]. In antegrade flow, the contrast will move in the normal direction of blood flow, from the internal carotid artery branching into the MCA and onwards. In retrograde flow, the contrast agent appears to move in the opposite direction, indicating a reversal of flow due to obstruction or significant decrease in the proximal pressure [33, 34].

The study’s data reflect a broad range of patient ages, correlating older age with more advanced stages of MMD (*p*-value = 0.045), thereby underscoring the progressive nature of the disease [35, 36]. The mean age of study patients was 47.03 years, which aligns with research from China [37], Korea [38], and Japan [39]. The observed correlation between older age and advanced Suzuki stages in MMD highlights the need for focused research on age-specific intervention efficacy and its impact on disease progression.

Our findings advocate for a paradigm shift in the surgical management of MMD, emphasizing the need for a hemodynamic evaluation over purely angiographic assessments. Such a shift could significantly influence surgical planning and outcomes, underscoring the importance of personalized treatment strategies that consider the complex interplay of hemodynamic factors in MMD.

Finally, this study was limited in that it was conducted in a single center with a limited sample size with no follow-up data as the aim was to explore, for the first time, the cortical perfusion pressure alteration pre- and post-bypass. Further research with larger cohorts of patients using multi-assessment tools is required to fully comprehend the impact of different donor-recipient pressure gradient on the on long-term outcomes in MMD patients.

## Conclusion

Our study elucidated that cerebral blood pressure significantly decreases beyond the moyamoya network at the distal M4 segment. Furthermore, we observed bidirectional flow in MCA territories, and maintaining a high-pressure gradient between donor and recipient arteries in STA-MCA bypass surgeries is achieved by selecting the downstream segment of the recipient artery for the anastomosis. The lack of correlation between Suzuki stages and M4 pressures indicates that angiographic severity may not reflect hemodynamic conditions before surgery. This highlights the need for a personalized hemodynamic evaluation and customized surgical approaches.

## Data Availability

The datasets during and/or analyzed during the current study available from the corresponding author on reasonable request.

## Abbreviations

DSA: Digital subtraction angiography
ECA: External carotid artery
ICA: Internal carotid artery
IQR: Interquartile range
IRB: Institutional Review Board
MAP: Mean arterial pressure
MCA: Middle cerebral artery
MMD: Moyamoya disease
STA: Superficial temporal artery

## References

[1] Research Committee on the Pathology and Treatment of Spontaneous Occlusion of the Circle of Willis, Health Labour Sciences Research Grant for Research on Measures for Infractable Diseases (2012). Guidelines for diagnosis and treatment of moyamoya disease (spontaneous occlusion of the circle of Willis). Neurol Med Chir (Tokyo).

[2] Scott RM, Smith ER (2009). Moyamoya disease and moyamoya syndrome. N Engl J Med..

[3] Burke GM, Burke AM, Sherma AK, Hurley MC, Batjer HH, Bendok BR (2009). Moyamoya disease: a summary. Neurosurg Focus..

[4] Guey S, Tournier-Lasserve E, Hervé D, Kossorotoff M (2015). Moyamoya disease and syndromes: from genetics to clinical management. Appl Clin Genet..

[5] Codd PJ, Scott RM, Smith ER (2009). Seckel syndrome and moyamoya. J Neurosurg Pediatr..

[6] Takeuchi K, Shimizu K (1957). Hypogenesis of bilateral internal carotid arteries. Brain and Nerve / No to Shinkei..

[7] Czabanka M, Peña-Tapia P, Schubert GA, Heppner FL, Martus P, Horn P (2011). Proposal for a new grading of moyamoya disease in adult patients. Cerebrovasc Dis..

[8] Hishikawa T, Hiramatsu M, Tokunaga K, Sugiu K, Date I. Pathophysiology of moyamoya disease. Jpn J Neurosurg. 2015;24:239–43. 10.7887/JCNS.24.239.

[9] Ravindra VM, Kralik SF, Griauzde J, Gadgil N, LoPresti MA, Lam S (2020). Preoperative computed tomography perfusion in pediatric moyamoya disease: a single-institution experience. J Neurosurg Pediatr..

[10] Li J, Jin M, Sun X, Li J, Liu Y, Xi Y (2019). Imaging of moyamoya disease and moyamoya syndrome: current status. J Comput Assist Tomogr..

[11] Suzuki J, Takaku A (1969). Cerebrovascular “moyamoya” disease. disease showing abnormal net-like vessels in base of brain. Arch Neurol.

[12] Kim SJ, Son TO, Kim KH, Jeon P, Hyun SH, Lee K-H (2014). Neovascularization precedes occlusion in moyamoya disease: angiographic findings in 172 pediatric patients. Eur Neurol..

[13] Sun H, Li W, Xia C, Ren Y, Ma L, Xiao A (2022). Angiographic and hemodynamic features in asymptomatic hemispheres of patients with moyamoya disease. Stroke..

[14] Lee JK, Williams M, Jennings JM, Jamrogowicz JL, Larson AC, Jordan LC (2013). Cerebrovascular autoregulation in pediatric moyamoya disease. Paediatr Anaesth..

[15] Ogawa A, Nakamura N, Yoshimoto T, Suzuki J (1990). Cerebral blood flow in moyamoya disease. Part 2: autoregulation and CO2 response. Acta Neurochir (Wien).

[16] Rosi A, Riordan CP, Smith ER, Scott RM, Orbach DB (2019). Clinical status and evolution in moyamoya: which angiographic findings correlate?. Brain Commun..

[17] Hussein AE, Brunozzi D, Shakur SF, Ismail R, Charbel FT, Alaraj A (2018). Cerebral aneurysm size and distal intracranial hemodynamics: an assessment of flow and pulsatility index using quantitative magnetic resonance angiography. Neurosurgery..

[18] Sun H, Tian R, Yu Z, Xiao A, You C, Liu Y (2021). Clinical and hemodynamic features in moyamoya disease with intracranial aneurysms. World Neurosurg..

[19] Helmy M, Liao Y, Luo S, Xu B. How I do it: direct pressure measurement in moyamoya bypass. Acta Neurochir. 2023;165(12):3631–5. 10.1007/s00701-023-05842-w. 10.1007/s00701-023-05842-w 37870662

[20] Holmes AP, Ray CJ, Kumar P, Coney AM (2020). A student practical to conceptualize the importance of Poiseuille’s law and flow control in the cardiovascular system. Adv Physiol Educ..

[21] Fujimura M, Sonobe S, Nishijima Y, Niizuma K, Sakata H, Kure S, et al. Genetics and biomarkers of moyamoya disease: significance of*RNF213*as a susceptibility gene. Journal of Stroke. 2014;16. 10.5853/jos.2014.16.2.65. Cited 2023 Sep 30. 10.5853/jos.2014.16.2.65 PMC4060268 24949311

[22] Phi JH, Wang K-C, Lee JY, Kim S-K (2015). Moyamoya syndrome: a window of moyamoya disease. J Korean Neurosurg Soc..

[23] Zhao MY, Armindo RD, Gauden AJ, Yim B, Tong E, Moseley M, et al. Revascularization improves vascular hemodynamics - a study assessing cerebrovascular reserve and transit time in Moyamoya patients using MRI. Journal of Cerebral Blood Flow and Metabolism: Official Journal of the International Society of Cerebral Blood Flow and Metabolism. 2023;43(2_suppl):138–51. 10.1177/0271678X221140343. 10.1177/0271678X221140343 PMC10638998 36408536

[24] Mount CA, M Das J. Cerebral perfusion pressure. StatPearls. Treasure Island (FL): StatPearls Publishing; 2023. http://www.ncbi.nlm.nih.gov/books/NBK537271/. Cited 2023 Sep 30. 30725956

[25] Miyamoto S, Yoshimoto T, Hashimoto N, Okada Y, Tsuji I, Tominaga T (2014). Effects of extracranial-intracranial bypass for patients with hemorrhagic moyamoya disease: results of the Japan Adult Moyamoya Trial. Stroke..

[26] Nielsen TH, Abhinav K, Sussman ES, Han SS, Weng Y, Bell-Stephens T (2020). Direct versus indirect bypass procedure for the treatment of ischemic moyamoya disease: results of an individualized selection strategy. J Neurosurg..

[27] Shulgina A, Lukshin V, Usachev D, Shevchenko E (2019). Local cerebral hemodynamics after superficial temporal artery-middle cerebral artery bypass in patients with symptomatic carotid occlusions. Asian J Neurosurg..

[28] Morisawa H, Kawamata T, Kawashima A, Hayashi M, Yamaguchi K, Yoneyama T (2013). Hemodynamics and changes after STA-MCA anastomosis in moyamoya disease and atherosclerotic cerebrovascular disease measured by micro-Doppler ultrasonography. Neurosurg Rev..

[29] Erdal AC, Gençpinar T. Chapter 20 - Biomechanics of circulation. In: Angin S, Şimşek IE, editors. Comparative Kinesiology of the Human Body. Academic Press; 2020. p. 365–71. https://www.sciencedirect.com/science/article/pii/B9780128121627000205. Cited 2024 Apr 7.

[30] Yu-jun LIAO, Kang-min HE, Bin XU (2022). Analysis of middle cerebral artery blood flow in moyamoya disease and comparison of orthodromic and antidromic bypass. Chin J Contemp Neurol Neurosurg.

[31] Lin M, Marshall CT, Qi Y, Johnston SM, Badea CT, Piantadosi CA (2009). Quantitative blood flow measurements in the small animal cardiopulmonary system using digital subtraction angiography. Medical Physics..

[32] Bonnefous O, Pereira VM, Ouared R, Brina O, Aerts H, Hermans R (2012). Quantification of arterial flow using digital subtraction angiography. Medical Physics..

[33] Iijima T, Mies G, Hossmann K-A. Repeated negative DC deflections in rat cortex following middle cerebral artery occlusion are abolished by MK-801: effect on volume of ischemic injury. Journal of Cerebral Blood Flow & Metabolism. 1992;12. 10.1038/jcbfm.1992.103. Cited 2024 Apr 5. 10.1038/jcbfm.1992.103 1506440

[34] Du L, Jiang H, Li J, Duan T, Zhou C, Yan F (2022). Imaging methods for surgical revascularization in patients with moyamoya disease: an updated review. Neurosurg Rev..

[35] Fujimura M, Tominaga T (2015). Current status of revascularization surgery for moyamoya disease: special consideration for its “internal carotid-external carotid (IC-EC) conversion” as the physiological reorganization system. Tohoku J Exp Med..

[36] Kim S-K, Seol HJ, Cho B-K, Hwang Y-S, Lee DS, Wang K-C (2004). Moyamoya disease among young patients: its aggressive clinical course and the role of active surgical treatment. Neurosurgery..

[37] Zhang D, Huang L, Huang Z, Zhou Q, Yang X, Gu H, et al. Epidemiology of moyamoya disease in China: a nationwide hospital-based study. The Lancet Regional Health – Western Pacific . 2022;18. https://www.thelancet.com/journals/lanwpc/article/PIIS2666-6065(21)00240-6/fulltext#%20. Cited 2023 May 21.

[38] Kim JS (2016). Moyamoya disease: epidemiology, clinical features, and diagnosis. J Stroke..

[39] Hoshino H, Izawa Y, Suzuki N, Disease RC on M (2012). Epidemiological features of moyamoya disease in Japan. Neurologia medico-chirurgica.

